# Population-Based Study of the Changes in the Food Choice Determinants of Secondary School Students: Polish Adolescents’ COVID-19 Experience (PLACE-19) Study

**DOI:** 10.3390/nu12092640

**Published:** 2020-08-30

**Authors:** Dominika Głąbska, Dominika Skolmowska, Dominika Guzek

**Affiliations:** 1 Department of Dietetics, Institute of Human Nutrition Sciences, Warsaw University of Life Sciences (SGGW-WULS), 159C Nowoursynowska Street, 02-776 Warsaw, Poland; dominika_skolmowska@sggw.edu.pl; 2 Department of Food Market and Consumer Research, Institute of Human Nutrition Sciences, Warsaw University of Life Sciences (SGGW-WULS), 159C Nowoursynowska Street, 02-776 Warsaw, Poland; dominika_guzek@sggw.edu.pl

**Keywords:** food choice, determinants, food choice questionnaire (FCQ), coronavirus-19, COVID-19, SARS-CoV-2, adolescents, national population-based study, PLACE-19 Study

## Abstract

During the outbreak of Coronavirus Disease 2019 (COVID-19) pandemic and the lockdown, various changes of dietary habits are observed, including both positive and negative ones. However, the food choice determinants in this period were not studied so far for children and adolescents. The study aimed to analyze the changes in the food choice determinants of secondary school students in a national sample of Polish adolescents within the Polish Adolescents’ COVID-19 Experience (PLACE-19) Study population. The study was conducted in May 2020, based on the random quota sampling of schools (for voivodeships and counties) and a number of 2448 students from all the regions of Poland participated. The Food Choice Questionnaire (FCQ) (36 items) was applied twice—to analyze separately current choices (during the period of COVID-19 pandemic) and general choices (when there was no COVID-19 pandemic). For both the period before and during the COVID-19 pandemic, sensory appeal and price were indicated as the most important factors (with the highest scores). However, differences were observed between the scores of specific factors, while health (*p* < 0.0001) and weight control (*p* < 0.0001) were declared as more important during the period of COVID-19 pandemic, compared with the period before, but mood (*p* < 0.0001) and sensory appeal (*p <* 0.0001) as less important. The observations were confirmed for sub-groups, while female and male respondents were analyzed separately. It can be concluded that the COVID-19 pandemic may have changed the food choice determinants of Polish adolescents, as it may have increased the importance of health and weight control, but reduced the role of mood and sensory appeal. This may be interpreted as positive changes promoting the uptake of a better diet than in the period before the pandemic.

## 1. Introduction

During the outbreak of Coronavirus Disease 2019 (COVID-19) caused by the spread of SARS-COV-2 virus, which was announced by the World Health Organization (WHO) as a pandemic on 11 March 2020 [1], there are novel problems arising in the globe which must be addressed in public health policy, including those associated with food and nutrition. Recent studies indicate that nutrition is becoming a more important issue than before because malnutrition [2] as well as obesity [3] may influence the outcomes of COVID-19 patients, and hence, high nutritional status should be maintained by following a properly balanced diet. At the same time, weight control is commonly stated as a problem during the COVID-19 lockdown, due to reduced physical activity and following an improperly balanced diet [4]. Taking this into account, the importance of following a properly balanced healthy diet is emphasized by major authorities, including the WHO [5], as it is indicated to potentially be significant not only for prevention but also for therapy, while the need for dedicated nutritional recommendations is considered as a priority [6].

A number of studies showed that the period of COVID-19 lockdown influenced the nutritional behaviors of the studied populations, while a lot of them analyzed the quality of diet of the subjects. They commonly indicated that, depending on the studied group, the influence of COVID-19 differed. For instance, such findings were reported in the study by Deschasaux-Tanguy et al. [7], which was conducted on a population of 37,252 French adults from the NutriNet-Sante cohort. The authors showed that in various groups of subjects, dietary behaviors were either improved or worsened. On the other hand, the study by Scarmozzino and Visioli [8] indicated that nearly half of the respondents did not substantially modify their dietary habits during the lockdown. In the study by Sidor and Rzymski [9], it was stated that in Poland, the body mass of adults was affected by their dietary habits, namely less frequent consumption of vegetables, fruits, and legumes during the COVID-19 quarantine. Similarly, the study conducted by Pietrobelli et al. [10] in a group of obese children showed that during the lockdown, the intake of unhealthy food such as potato chips, red meat, and sugary drink was increased.

Food choices made by a person during adolescence are especially important because the intake of food in this period may determine both the current health status and the health status in adult life [11]. Food choice determinants of adolescents have been shown to be complex and multicausal. The study of Share and Stewart-Knox [12] conducted in a group of Irish adolescents distinguished 5 motivation factors—health, price/convenience, mood, religion, and animal rights. In an American study by Neumark-Sztainer et al. [13], both internal and external factors influencing the food choices of adolescents were discerned, which included the appeal of food, food availability, mood, body image, parental influences, and media. While some of the factors perceived as important in choosing certain types of food are permanent and influence food choices for the whole life, others are specifically associated with being an adolescent [14].

Although a number of studies have revealed that the period of COVID-19 pandemic influenced the dietary behaviors of respondents [7,8,9,10], their food choice motivations still remain poorly understood. Some studies have also evaluated specific food product choices. For example, in the study by Jribi et al. [15], a majority of respondents stated that COVID-19 lockdown modified their grocery shopping habits and improved their shopping performance. Similarly, in the study by Di Renzo et al. [16], several respondents declared buying organic food or directly from farmers, as well as purchasing more fruits and vegetables. Additionally, the study of Bracale and Vaccaro [17] showed changes in the food choices of people in the period of COVID-19, with an increase in the consumption of flour, pasta, eggs, and frozen food and a decrease in the consumption of fresh food. However, no studies published so far analyzed the factors that determined food choice determinants during the COVID-19 pandemic.

For both adults [18] and children [19], numerous determinants of food choices and eating behaviors have been identified. The period of COVID-19 pandemic may have modified the existing determinants, as during this period people experienced a number of stressors, such as prolonged duration of lockdown, fears of infection, frustration, and boredom, inadequate information, lack of personal space at home, and family financial loss, as well as lack of in-person contact with classmates, friends, and teachers, which in the case of children and adolescents may influence the dietary habits [20]. Therefore, there is a need to analyze in detail the food choice determinants of adolescents, as this knowledge will be particularly relevant to nutritionists, educators, policymakers, and manufacturers who are interested in the nutritional status and well-being of adolescents.

Taking this into account, the present study aimed to analyze the changes in the food choice determinants of secondary school students in a national sample of Polish adolescents within the Polish Adolescents’ COVID-19 Experience (PLACE-19) Study population.

## 2. Materials and Methods

### 2.1. PLACE-19 Study Population

The presented study was conducted in a sample of Polish secondary school students within the second phase of the PLACE-19 Study. The first phase was conducted in April 2020 and was associated with the hand hygiene behaviors and other protective measures that were applied in this period [21,22]. The second phase was associated with nutritional behaviors and was conducted in the period of May 2020 (1st stage—from 29 April 2020 to 10 May 2020 and 2nd stage—from 11 May 2020 to 23 May 2020). The study was conducted on a national sample of Polish respondents according to the guidelines laid down in the Declaration of Helsinki, and all procedures involving human subjects were approved by the Ethics Committee of the Institute of Human Nutrition Sciences of the Warsaw University of Life Sciences (no. 20/2020). All participants provided their informed consent to participate in the study.

The study was based on the random quota sampling of secondary schools that was conducted to invite the representative population of schools from each region of country. Poland is divided into 6 main geographic regions with 16 administrative units (voivodeships), depending on historic, cultural, economic, and geographic factors, and each voivodeship is further divided into counties. The stratified sampling of secondary schools was conducted and they were randomly chosen to be invited to participate in the study, based on (1) the sampling of counties within voivodeships (5 counties for each of 16 voivodeships of Poland—total number of 80 counties), followed by (2) sampling of secondary schools within counties (10 schools for each of 80 counties—total number of 800 schools), within 1st stage. The identical supplementary sampling was conducted for those voivodeships for which there was insufficient number of respondents after 10 days of data gathering, based on (1) the sampling of counties within voivodeships (5 counties for each of 8 voivodeships of Poland—total number of 40 counties), followed by (2) sampling of secondary schools within counties (10 schools for each of 40 counties—total number of 400 schools), within 2nd stage. The total number of schools that were invited to participate in the study was 1200, while the schools that were included to the first phase of the study (conducted in April 2020), were excluded from the sampling to the second phase. The local Boards of Education were engaged, if needed, to arrange the study.

The Head of each secondary school, that was sampled within the procedure, was invited for the participation of school in the study, while the participation was voluntary. If they expressed the will for the school to participate in the study, they informed students that they are invited to participate, while their participation was also voluntary. The students were included only if they, as well as their parents/legal guardians, provided informed consent to participate. If so, they were provided an electronic link to the dedicated questionnaire. The questionnaire form was anonymized and did not allow to gather any data that would allow to identify the respondent. Additionally, the questionnaire form did not include any questions about such issues that may be perceived as personal or sensitive ones. Due to the fact that some Heads of secondary schools did not provide information if they would like to participate or not, after one week of the 1st stage and of the 2nd stage, they received a reminder.

The students of each secondary school were informed that a link to the questionnaire is dedicated only for them, as the study was conducted only in a specific sample of students from the randomly chosen schools, who provided their informed consent to participate and those aged 15–20 years (being a typical age for this level of education in Poland) (inclusion criteria). The respondents of the study were excluded if they provided their questionnaires with any missing/unreliable data.

The number of included participants was 2448. As in Poland the vast majority of adolescents aged 15–20 years are the secondary school students (the current Net Enrollment Rate (NER) is calculated as 89.38%, based on the statistics of the Central Statistical Office (CSO) in Poland based on data for December 2019 [23]), the included sample was considered population-based.

**Figure 1:**
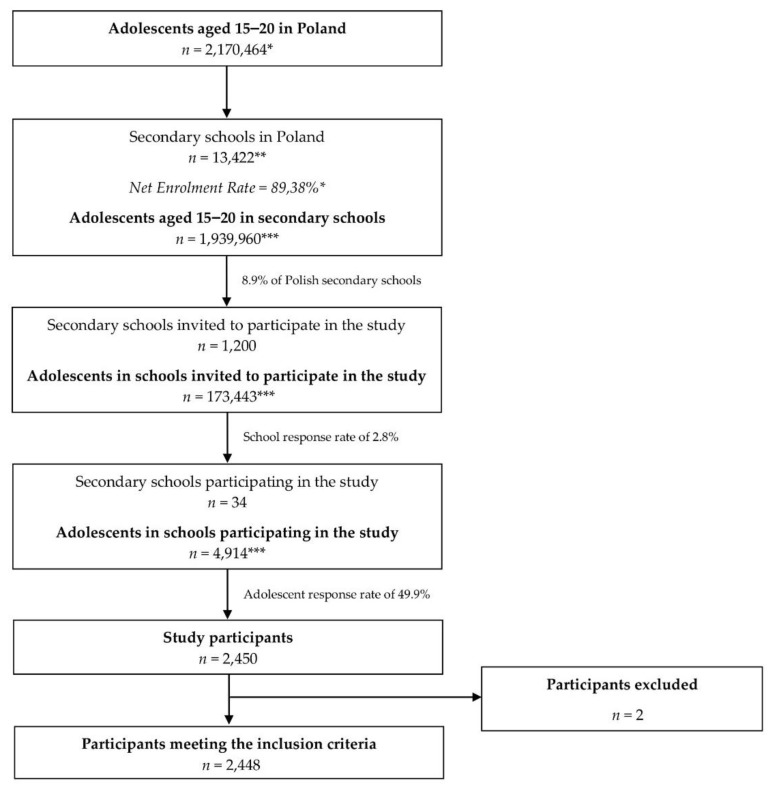
The procedure of secondary school sampling and students including to the Polish Adolescents’ COVID-19 Experience (PLACE-19) Study. * statistics of the Central Statistical Office (CSO) in Poland [23,24]; ** statistics of the Polish Ministry of National Education [25]; *** calculated on the basis of the statistics of CSO.

The procedure of secondary school sampling and students including to the PLACE-19 Study is presented in Figure 1.

### 2.2. Food Choice Questionnaire (FCQ)

The study was conducted based on the data collected from a group of secondary school students within the PLACE-19 Study. Information about food choice determinants and changes was obtained from the subjects by using the Food Choice Questionnaire (FCQ), developed by Steptoe et al. [26]. This questionnaire is commonly applied in various populations [27,28], including adolescents [29,30].

The FCQ was designed to assess a wide range of considerations that individuals might have when choosing what to eat, in order to evaluate the motives underlying people’s selection of food products and dishes divided into 9 categories as health, mood, convenience, sensory appeal, natural content, price, weight control, familiarity, and ethical concern [26]. Within each category, specific items are defined, and respondents were asked how important they considered an item while choosing the food they eat on a typical day.

In the study, the respondents were asked using the same FCQ to indicate their food product choices twice, but focusing separately on their current choices (during the period of COVID-19 pandemic) and on their general choices (when there was no COVID-19 pandemic). As it may have been hard for them to discriminate the before and current period of COVID-19 pandemic, they were asked about the current period of their remote education and separately about the period before their remote education. This is because, in the period of the study, students were receiving remote education (since 12 March 2020, as decided by the Polish Ministry of National Education [31]), after the first COVID-19 case was diagnosed (4 March 2020 [32]). As FCQ is a tool with proven reproducibility (2- to 3-week period) [24], the differences observed while comparing the period before and during the COVID-19 pandemic may be attributed to differences in the food choice determinants caused by the pandemic.

The FCQ applied in the study, which was developed by Steptoe et al. [26], was based on 36 items. For each item, the respondents were asked to endorse the statement, “It is important to me that the food I eat on a typical day…”, by choosing between one of the following 4 responses (close-ended question): not at all important (scored as 1), a little important (scored as 2), moderately important (scored as 3), and very important (scored as 4). Afterward, the items were clustered within 9 factors, as described by Steptoe et al. [26]: health (6 items included), mood (6 items included), convenience (5 items included), sensory appeal (4 items included), natural content (3 items included), price (3 items included), weight control (3 items included), familiarity (3 items included), and ethical concern (3 items included). Finally, for each respondent, each factor, as a determinant of food choices, was scored with the mean score of the items included. The results obtained for each factor (9 factors), as well as for each item within a factor (36 items), were compared for the period before and during the COVID-19 pandemic, in order to perform an in-depth analysis, as applied by other authors [33,34].

### 2.3. Statistical Analysis

To verify the internal reliability of data within assessed factors, the Cronbach’s alpha coefficient was applied [35]. The Shapiro-Wilk test was applied to verify the normality of distribution of data. The Principal Component Analysis (PCA), based on the analysis of correlations, was applied to define the loadings for the items included within the factors. The results obtained for the period before the COVID-19 pandemic and during the COVID-19 pandemic were compared while using Wilcoxon matched pair *T*-test (due to nonparametric distributions), accompanied by effect size verification (only the results with *r* ≥ 0.15 were discussed, as according to Cohen’s classification of effect sizes *r* < 0.1 represents no effect [36]).

The *p* ≤ 0.05 level was interpreted as statistically significant. The statistical analysis was conducted while using Statistica version 13.3 (StatSoft Inc., Tulsa, OK, USA).

## 3. Results

The general characteristics of the sample of adolescents studied within the PLACE-19 Study is presented in Table 1. The study was conducted in a homogenic sample of Polish adolescents, while mainly female students participated.

The results of the loading and Cronbach’s alpha coefficient for the applied FCQ for the period before and during the COVID-19 pandemic for the sample of adolescents studied within the PLACE-19 Study are presented in Table 2. Both for the period before and during the COVID-19 pandemic, the Cronbach’s alpha coefficients for the analyzed factors indicated a good internal reliability of the data. The loading of the specific items was similar in the period before and during the COVID-19 pandemic.

The results of the loading and Cronbach’s alpha coefficient for the applied FCQ for the period before and during the COVID-19 pandemic for the sample of female adolescents studied within the PLACE-19 Study are presented in Table 3. Both for the period before and during the COVID-19 pandemic, the Cronbach’s alpha coefficients for the analyzed factors indicated a good internal reliability of the data. The loading of the specific items was similar in the period before and during the COVID-19 pandemic. Moreover, the results were similar as for the general studied population.

The results of the loading and Cronbach’s alpha coefficient for the applied FCQ for the period before and during the COVID-19 pandemic for the sample of male adolescents studied within the PLACE-19 Study are presented in Table 4. Both for the period before and during the COVID-19 pandemic, the Cronbach’s alpha coefficients for the analyzed factors indicated a good internal reliability of the data. The loading of the specific items was similar in the period before and during the COVID-19 pandemic. Moreover, the results were similar as for the general studied population.

The factors influencing food choices determined based on the applied FCQ for the period before and during the COVID-19 pandemic in the sample of adolescents studied within the PLACE-19 Study are presented in Table 5. The additional analyses of the results for all the items included within the factors of the applied FCQ are presented in Supplementary Tables (Tables S1–S9). For both the period before and during the COVID-19 pandemic, sensory appeal and price were indicated as the most important factors (with the highest scores) by the respondents. However, differences were observed between the scores of specific factors, while health (*p* < 0.0001) and weight control (*p* < 0.0001) were declared as more important during the period of COVID-19 pandemic, compared with the period before, but mood (*p* < 0.0001) and sensory appeal (*p <* 0.0001) as less important.

The factors influencing food choice based on the applied FCQ for the period before and during the COVID-19 pandemic for the sample of female adolescents studied within the PLACE-19 Study are presented in Table 6. Both for the period before and during the COVID-19 pandemic, the sensory appeal and price were indicated as the most important factors (with the highest scores). However, there were differences between the scores of specific factors, while the results were similar as for the general studied population. The health (*p* < 0.0001) and weight control (*p* < 0.0001) in the period of COVID-19 pandemic were assessed as more important, while compared with the period before, but mood (*p* = 0.0002), and sensory appeal (*p <* 0.0001) as less important.

The factors influencing food choice based on the applied FCQ for the period before and during the COVID-19 pandemic for the sample of male adolescents studied within the PLACE-19 Study are presented in Table 7. Both for the period before and during the COVID-19 pandemic, the sensory appeal and price were indicated as the most important factors (with the highest scores). However, there were differences between the scores of specific factors, while the results were similar as for the general studied population. The weight control (*p* < 0.0001) in the period of COVID-19 pandemic was assessed as more important, while compared with the period before, but mood (*p* < 0.0001), and sensory appeal (*p <* 0.0001) as less important.

## 4. Discussion

The conducted study revealed some important differences in the food choice determinants (described in the FCQ as factors) for the period before and during the COVID-19 global pandemic, as the importance given to health and weight control increased, while that of mood and sensory appeal decreased during the COVID-19 pandemic. The observations were confirmed for sub-groups, while female and male respondents were analyzed separately.

Taking into account the significance of following a properly balanced diet during the COVID-19 pandemic, prioritizing the food choice determinants associated with health and weight control should be perceived as beneficial. However, it should be mentioned that among the items associated with health, the change considered as the most significant was the fiber content, but not the content of vitamins or minerals, or keeping healthy. It may be explained by the general connotation that food products high in fiber are low in calories [37]. At the same time, the reason may be associated with the sedentary lifestyle and resulting constipation or problems in bowel movements [38], which may be reduced by increased fiber intake [39].

Simultaneously, for weight control as a determinant of food choices in the period of COVID-19 pandemic, all the assessed items were declared as more important than before, including those associated with energy value, fat content, and weight control potential. This indicated the major role of all the described aspects in managing the individual diet of respondents, which is in agreement with the priorities of the European Association for the Study of Obesity [40]. Analysis of the global results on the problems with maintaining a stable body mass during COVID-19 lockdown [41] showed that these may be associated with the gain of body mass during the initial period of lockdown and the resultant need to reduce the weight.

For the food choice determinants perceived as less important during the COVID-19 pandemic than before, including mood and sensory appeal, it should be emphasized that not considering them when choosing food may be interpreted as beneficial in terms of following a properly balanced diet. Moreover, among the items associated with mood, only the importance of helping to cope with life increased, while it cannot be unambiguously interpreted as having any dietary restrictions and as resulting from the temporary urge. At the same time, as mood is a determinant that mainly influences the increased consumption of sweets and fatty food of high energy density, which may mitigate the effects of stress on opioidergic and dopaminergic neurotransmission in the brain [42], its decreased role may promote the intake of a diet of higher nutritional value.

To our knowledge, this is the first study conducted during the COVID-19 pandemic to determine the food choice determinants of the adolescents in this unusual period using FCQ. However, FCQ is a reliable tool which was already applied in various populations, including adolescents. Therefore, the observations of the present study may be compared to those from other studies that were conducted while there was no COVID-19 pandemic. In the study of Canales et al. [29], which was conducted in a group of Spanish high school students, it was indicated that adolescents chose their food based on sensory appeal and price, while ethical concern was the least important for them. The study of Maulida et al. [30] conducted in a group of junior high school students from Indonesia showed that students from less affluent families put more emphasis on price and convenience in terms of food choices, while those from more affluent families did not make healthier food choices.

Similar determinants have been declared by adult populations in different studies; for example, manufacturing workers in Brazil indicated sensory appeal and price as the most important determinants [43]; relatives of secondary and high school students in Spain indicated sensory appeal, price, and weight control [44]; adults from Western Balkan countries indicated sensory appeal, convenience, health, and natural content [45]; individuals from Belgium, Hungary, and Romania indicated sensory appeal, health, convenience, and price; and individuals from the Philippines indicated health, price, mood, and sensory appeal [27]. In the comprehensive study of Markovina conducted in 9 European countries, price was found to be the most important determinant of food choices in Spain, Greece, Ireland, Portugal, and the Netherlands, sensory appeal in Norway, Germany, and the United Kingdom, and natural content in Poland, while familiarity and ethical concern were consistently ranked as the least important by the populations of all the studied countries [46]. At the same time, the systematic review of cross-cultural and single-country studies by Cunha et al. [47] showed that sensory appeal was considered as the most important determinant by consumers from Bosnia-Herzegovina, Belgium, Canada, Germany, Croatia, Hungary, Montenegro, Republic of Macedonia, Norway, New Zealand, Romania, Serbia, Slovenia, and the United Kingdom, followed by health and price, while ethical concern and familiarity were ranked as the least important determinants. Comparing the results of our study with those presented above, it can be stated that sensory appeal and price are important determinants of food choices, independent of the studied group, and that the subjects considered them as the most important factors both before and during the COVID-19 pandemic.

As the presented results were obtained by using the commonly applied and valid questionnaire [47], in a national sample of adolescents, gathered by sampling in all the regions, the findings of our study may be significant for further public health policy. Moreover, as no such studies were conducted so far in other countries, in this specific period of COVID-19 pandemic, the results may be extrapolated to other countries. However, it must be pointed out that the results described for the period before the COVID-19 global pandemic were based on reporting at the time of the pandemic, so there may be a possibility of recall bias which cannot be excluded.

The broad implications from the present study should be listed, as well as highlighting the possibilities of using the obtained results is needed. It must be specified that the food choice determinants, while changing during any long-term crisis (as a prolonged pandemic), may also interfere with other areas, including social, economic, and environmental. The social practices and social norms include nutritional aspects, so while health and weight control were considered as more important during the pandemic than before, they may influence the general social interactions, including social stigmatization and prejudice and thus result in marginalization and inequities against individuals with other food choice priorities. Changes in food choice determinants may influence the demand for food products, as products that have health-promoting properties, are low in calories, or are low in fat may be expected to be perceived as more valuable, increasing the market pressure on producers. Last but not least, the general situation may change the structure of consumption, influencing the national and international markets, the environmental impact of production, and the necessary actions that should be taken by governments. It must be indicated that evolving food choice determinants, which were observed in the case of Polish adolescents, while being associated with positive effects during the COVID-19 pandemic, may also be perceived as an opportunity to improve the eating behaviors of people and create more beneficial dietary habits. Taking this into account, governments within their public health policy should specify the positive behaviors that may be adopted by consumers to improve their general well-being, mood, and health.

It is well known that various factors influence the food choices of consumers, including sensory appeal, price, and health concerns [47]. Understanding these factors may contribute to promoting the consumption of healthy food products and limitation of those not having health benefits [48]. Knowledge about food choice determinants, their changes, and association with the current global situation will allow designing appropriate and effective dietary policies aimed at specific populations, bearing in mind the food-related customs, which are particularly associated with the cultural background [49]. This is especially important, as the COVID-19 pandemic has been indicated to be associated with changes in eating habits, including increased consumption of homemade dishes, such as pizza, bread, and sweets, and decreased intake of fresh fish and packed sweets [16]. Therefore, identifying the motives for choosing certain types of food among adolescents will be helpful to establish successful educational campaigns which will be needed soon.

The other issue that must be addressed, especially in terms of necessary national and international policies, is the fact that changes observed in food choice determinants may be either sustained in the future or only temporary. Without further observation after the COVID-19 pandemic, we will not understand if this global crisis will change the general approach, as well as the individual values and priorities of populations once and for all.

Although the present study provided novel information about food choice determinants in the population of adolescents during the COVID-19 pandemic, it has some limitations that must be highlighted. The most important is that the study may be associated with general recall bias, resulting from chances of inaccurate and incomplete recollection of events by the respondents. However, as the COVID-19 pandemic was an unexpected event, it was impossible to conduct a prospective study to verify its influence, which would be the only way to overcome this bias. Taking this into account, as well as considering that no studies have been published so far analyzing the food choice determinants during the COVID-19 pandemic, it may be stated that the presented study showed some valuable results.

The other limitations are associated with the fact that the study was conducted only in the population of one country, and although it provided detailed information about this specific population, it should be reproduced in other countries as well. At the same time, it is crucial to analyze the food choice determinants in representative groups, and while in the present study a higher share of female than male individuals participated, it may be supposed to have influenced the representativeness of the studied group. Moreover, the study was based on anonymous and voluntary declarations, as it was conducted using an online survey; therefore, it presents only declarative determinants of food choices not verified in real market conditions. However, this limitation would be impossible to overcome, as many restrictions were implemented in Poland during the COVID-19 pandemic, including the lockdown. Last but not least, it must be emphasized that in Poland, adolescents rarely do household grocery shopping, so the analysis of their food choice determinants does not reveal the full picture of the situation.

## 5. Conclusions

In the study, it was observed that both before and during the COVID-19 pandemic, sensory appeal and price were considered as the most important factors determining food choices by Polish adolescents. However, the study also showed differences between the scores of specific factors, while health and weight control were declared as more important during the COVID-19 pandemic compared with the period before, but mood and sensory appeal as less important. Taking into account the period of COVID-19 pandemic, prioritizing the food choice determinants based on health and weight control should be perceived as beneficial and may be considered as a conscious decision needed for this period.

It can be concluded that the COVID-19 pandemic may have changed the food choice determinants of Polish adolescents, as it may have increased the importance of health and weight control but reduced the role of mood and sensory appeal. This may be interpreted as positive changes promoting the uptake of a better diet than in the period before the pandemic.

These findings, if confirmed by other studies, may be valuable while developing local and national nutritional strategies for Poland and other countries. It should be kept in mind that changes in food choice determinants may also influence the nutritional behaviors of people, so while promoting a healthy diet, the general situation must be reconsidered. Therefore, educational strategies addressed for children and adolescents, as well as for parents, to influence their nutritional behaviors, should consider the novel and unusual situation of the pandemic. Such education is necessary and required actions should be appropriately planned, as consumers seem to be more focused on their food choices and may thus seek nutritional knowledge to make more informed decisions. Especially in the period of COVID-19 global pandemic, general well-being should be the focus of all measures, and hence including nutritional education may be valuable for public health interventions.

## Figures and Tables

**Table 1:** The general characteristics of the sample of adolescents studied within the PLACE-19 Study (*n* = 2448).

Characteristics		Results
Age (years)	Mean ± SD	16.8 ± 1.1
	Median (range)	17 (15–20)
Gender	Female	1552 (63.4%)
	Male	896 (36.6%)

**Table 2:** The results of the loading and Cronbach’s alpha coefficient for the applied Food Choice Questionnaire (FCQ) for the period before and during the Coronavirus Disease 2019 (COVID-19) pandemic for the sample of adolescents studied within the PLACE-19 Study (*n* = 2448).

Descriptions of Items Defined within Food Choice Questionnaire (FCQ) *	Before the COVID-19 Pandemic		During the COVID-19 Pandemic	
	Loading	α **	Loading	α **
**Factor 1—Health**				
Contains a lot of vitamins and minerals	0.87	0.90	0.90	0.93
Is nutritious	0.86		0.90	
Keeps me healthy	0.84		0.86	
Is high in protein	0.79		0.81	
Is good for my skin/teeth/hair/nails etc.	0.79		0.82	
Is high in fiber and roughage	0.78		0.84	
**Factor 2—Mood**				
Helps me relax	0.84	0.84	0.87	0.90
Cheers me up	0.80		0.83	
Helps me to cope with life	0.77		0.83	
Helps me cope with stress	0.76		0.83	
Keeps me awake/alert	0.71		0.76	
Makes me feel good	0.58		0.74	
**Factor 3—Convenience**				
Takes no time to prepare	0.85	0.85	0.88	0.89
Is easily available in shops and supermarkets	0.73		0.80	
Can be cooked very simply	0.72		0.88	
Can be bought in shops close to where I live or work	0.68		0.80	
Is easy to prepare	0.62		0.81	
**Factor 4—Sensory Appeal**				
Smells nice	0.85	0.82	0.90	0.84
Looks nice	0.85		0.89	
Has a pleasant texture	0.74		0.77	
Tastes good	0.70		0.75	
**Factor 5—Natural Content**				
Contains no artificial ingredients	0.87	0.81	0.90	0.86
Contains natural ingredients	0.86		0.90	
Contains no additives	0.83		0.87	
**Factor 6—Price**				
Is cheap	0.87	0.80	0.90	0.87
Is not expensive	0.86		0.90	
Is good value for money	0.81		0.86	
**Factor 7—Weight Control**				
Is low in calories	0.88	0.82	0.91	0.87
Is low in fat	0.85		0.89	
Helps me control my weight	0.85		0.89	
**Factor 8—Familiarity**				
Is what I usually eat	0.82	0.65	0.87	0.78
Is familiar	0.77		0.85	
Is like the food I ate when I was a child	0.71		0.78	
**Factor 9—Ethical Concern**				
Has the country of origin clearly marked	0.85	0.78	0.89	0.84
Comes from countries I approve of politically	0.84		0.89	
Is packaged in an environmentally friendly way	0.80		0.83	

* FCQ as described by Steptoe et al. [26]; ** Cronbach’s alpha coefficient [35].

**Table 3:** The results of the loading and Cronbach’s alpha coefficient for the applied Food Choice Questionnaire (FCQ) for the period before and during the COVID-19 pandemic for the sample of female adolescents studied within the PLACE-19 Study (*n* = 1552).

Descriptions of Items Defined within Food Choice Questionnaire (FCQ) *	Before the COVID-19 Pandemic		During the COVID-19 Pandemic	
	Loading	α **	Loading	α **
**Factor 1—Health**				
Contains a lot of vitamins and minerals	0.87	0.91	0.92	0.92
Is nutritious	0.86		0.91	
Keeps me healthy	0.85		0.86	
Is high in protein	0.82		0.86	
Is good for my skin/teeth/hair/nails etc.	0.79		0.89	
Is high in fiber and roughage	0.80		0.86	
**Factor 2—Mood**				
Helps me relax	0.85	0.84	0.89	0.90
Cheers me up	0.80		0.83	
Helps me to cope with life	0.78		0.83	
Helps me cope with stress	0.77		0.84	
Keeps me awake/alert	0.69		0.75	
Makes me feel good	0.60		0.76	
**Factor 3—Convenience**				
Takes no time to prepare	0.86	0.85	0.88	0.89
Is easily available in shops and supermarkets	0.76		0.79	
Can be cooked very simply	0.85		0.88	
Can be bought in shops close to where I live or work	0.75		0.80	
Is easy to prepare	0.72		0.80	
**Factor 4—Sensory Appeal**				
Smells nice	0.85	0.79	0.90	0.85
Looks nice	0.85		0.89	
Has a pleasant texture	0.74		0.77	
Tastes good	0.69		0.76	
**Factor 5—Natural Content**				
Contains no artificial ingredients	0.83	0.80	0.86	0.86
Contains natural ingredients	0.85		0.89	
Contains no additives	0.85		0.90	
**Factor 6—Price**				
Is cheap	0.87	0.80	0.91	0.87
Is not expensive	0.86		0.91	
Is good value for money	0.80		0.86	
**Factor 7—Weight Control**				
Is low in calories	0.88	0.82	0.91	0.87
Is low in fat	0.84		0.88	
Helps me control my weight	0.85		0.88	
**Factor 8—Familiarity**				
Is what I usually eat	0.83	0.66	0.88	0.79
Is familiar	0.77		0.86	
Is like the food I ate when I was a child	0.71		0.78	
**Factor 9—Ethical Concern**				
Has the country of origin clearly marked	0.84	0.76	0.89	0.83
Comes from countries I approve of politically	0.83		0.89	
Is packaged in an environmentally friendly way	0.79		0.83	

* FCQ as described by Steptoe et al. [26]; ** Cronbach’s alpha coefficient [35].

**Table 4:** The results of the loading and Cronbach’s alpha coefficient for the applied Food Choice Questionnaire (FCQ) for the period before and during the COVID-19 pandemic for the sample of male adolescents studied within the PLACE-19 Study (*n* = 896).

Descriptions of Items Defined within Food Choice Questionnaire (FCQ) *	Before the COVID-19 Pandemic		During the COVID-19 Pandemic	
	Loading	α **	Loading	α **
**Factor 1—Health**				
Contains a lot of vitamins and minerals	0.86	0.89	0.89	0.91
Is nutritious	0.85		0.90	
Keeps me healthy	0.82		0.84	
Is high in protein	0.78		0.79	
Is good for my skin/teeth/hair/nails etc.	0.77		0.79	
Is high in fiber and roughage	0.75		0.81	
**Factor 2—Mood**				
Helps me relax	0.81	0.82	0.87	0.88
Cheers me up	0.79		0.82	
Helps me to cope with life	0.76		0.82	
Helps me cope with stress	0.72		0.80	
Keeps me awake/alert	0.72		0.76	
Makes me feel good	0.54		0.68	
**Factor 3—Convenience**				
Takes no time to prepare	0.84	0.85	0.88	0.89
Is easily available in shops and supermarkets	0.77		0.81	
Can be cooked very simply	0.84		0.82	
Can be bought in shops close to where I live or work	0.77		0.80	
Is easy to prepare	0.74		0.87	
**Factor 4—Sensory Appeal**				
Smells nice	0.85	0.79	0.87	0.83
Looks nice	0.85		0.88	
Has a pleasant texture	0.72		0.76	
Tastes good	0.71		0.73	
**Factor 5—Natural Content**				
Contains no artificial ingredients	0.82	0.80	0.86	0.85
Contains natural ingredients	0.85		0.88	
Contains no additives	0.86		0.89	
**Factor 6—Price**				
Is cheap	0.87	0.81	0.89	0.86
Is not expensive	0.86		0.90	
Is good value for money	0.83		0.86	
**Factor 7—Weight Control**				
Is low in calories	0.86	0.80	0.89	0.86
Is low in fat	0.85		0.88	
Helps me control my weight	0.83		0.87	
**Factor 8—Familiarity**				
Is what I usually eat	0.81	0.64	0.86	0.76
Is familiar	0.77		0.83	
Is like the food I ate when I was a child	0.71		0.78	
**Factor 9—Ethical Concern**				
Has the country of origin clearly marked	0.86	0.81	0.90	0.85
Comes from countries I approve of politically	0.87		0.88	
Is packaged in an environmentally friendly way	0.83		0.85	

* FCQ as described by Steptoe et al. [26]; ** Cronbach’s alpha coefficient [35].

**Table 5:** The factors influencing food choice based on the applied Food Choice Questionnaire (FCQ) for the period before and during the COVID-19 pandemic for the sample of adolescents studied within the PLACE-19 Study (*n* = 2448).

Factors	Before the COVID-19 Pandemic		During the COVID-19 Pandemic		*p*	*r*
	Median (IQR)	25th–75th	Median (IQR)	25th–75th		
**Health**	2.7 (1.3)	2.0–3.3	2.8 (1.5)	2.0–3.5	<0.0001	0.15
**Mood**	2.7 (1.2)	2.0–3.2	2.5 (1.2)	2.0–3.2	<0.0001	0.16
**Convenience**	2.8 (1.2)	2.2–3.4	2.8 (1.4)	2.0–3.4	0.0177	0.06
**Sensory Appeal**	3.1 (1.3)	2.5–3.8	3.0 (1.2)	2.3–3.5	<0.0001	0.29
**Natural Content**	2.7 (1.3)	2.0–3.3	2.7 (1.3)	2.0–3.3	0.0122	0.07
**Price**	3.0 (1.0)	2.3–3.3	3.0 (1.7)	2.0–3.7	0.0125	0.07
**Weight Control**	2.3 (1.3)	1.7–3.0	2.7 (1.3)	2.0–3.3	<0.0001	0.25
**Familiarity**	2.3 (1.0)	2.0–3.0	2.3 (1.3)	1.7–3.0	0.4473	0.02
**Ethical Concern**	2.0 (1.4)	1.3–2.7	2.0 (1.4)	1.3–2.7	<0.0001	0.13

IQR—interquartile range.

**Table 6:** The factors influencing food choice based on the applied Food Choice Questionnaire (FCQ) for the period before and during the COVID-19 pandemic for the sample of female adolescents studied within the PLACE-19 Study (*n* = 1552).

Factors	Before the COVID-19 Pandemic		During the COVID-19 Pandemic		*p*	*r*
	Median (IQR)	25th–75th	Median (IQR)	25th–75th		
**Health**	2.8 (1.3)	2.2–-3.5	3.0 (1.5)	2.2–3.7	<0.0001	0.19
**Mood**	2.8 (1.2)	2.2–3.3	2.7 (1.3)	2.0–3.3	0.0002	0.22
**Convenience**	2.8 (1.2)	2.2–3.4	2.8 (1.2)	2.2–3.4	0.0104	0.23
**Sensory Appeal**	3.3 (1.0)	2.8–3.8	3.0 (1.3)	2.5–3.8	<0.0001	0.16
**Natural Content**	2.7 (1.3)	2.0–3.3	3.0 (1.7)	2.0–3.7	0.0046	0.22
**Price**	3.0 (1.0)	2.3–3.3	3.0 (1.5)	2.2–3.7	0.0388	0.23
**Weight Control**	2.7 (1.3)	2.0–3.3	2.7 (1.7)	2.0–3.7	<0.0001	0.18
**Familiarity**	2.3 (1.0)	2.0–3.0	2.3 (1.3)	1.7–3.0	0.3097	0.24
**Ethical Concern**	2.0 (1.3)	1.3–2.7	2.0 (1.7)	1.3–3.0	0.0220	0.23

IQR—interquartile range.

**Table 7:** The factors influencing food choice based on the applied Food Choice Questionnaire (FCQ) for the period before and during the COVID-19 pandemic for the sample of male adolescents studied within the PLACE-19 Study (*n* = 896).

Factors	Before the COVID-19 Pandemic		During the COVID-19 Pandemic		*p*	*r*
	Median (IQR)	25th–75th	Median (IQR)	25th–75th		
**Health**	2.5 (1.2)	1.8–3.0	2.5 (1.0)	2.0–3.0	0.4063	0.24
**Mood**	2.3 (1.0)	2.0–3.0	2.3 (1.2)	1.8–3.0	<0.0001	0.18
**Convenience**	2.6 (1.2)	2.0–3.2	2.6 (1.2)	2.0–3.2	0.5046	0.24
**Sensory Appeal**	3.0 (1.1)	2.4–3.5	2.8 (1.0)	2.3–3.3	<0.0001	0.17
**Natural Content**	2.3 (1.3)	1.7–3.0	2.3 (1.3)	1.7–3.0	0.9661	0.25
**Price**	2.7 (1.3)	2.0–3.3	2.7 (1.3)	2.0–3.3	0.1908	0.23
**Weight Control**	2.0 (1.4)	1.3–2.7	2.0 (1.7)	1.3–3.0	<0.0001	0.19
**Familiarity**	2.3 (1.0)	2.0–3.0	2.3 (1.3)	1.7–3.0	0.9590	0.25
**Ethical Concern**	1.7 (1.3)	1.0–2.3	2.0 (1.7)	1.0–2.7	<0.0001	0.18

IQR—interquartile range.

## Supplementary Materials

The following are available online at https://www.mdpi.com/2072-6643/12/9/2640/s1: Table S1. The results for the items included within the Factor—Health of the applied Food Choice Questionnaire (FCQ) for the period before and during the COVID-19 pandemic for the sample of adolescents studied within the PLACE-19 Study (*n* = 2448). Table S2. The results for the items included within the Factor—Mood of the applied Food Choice Questionnaire (FCQ) for the period before and during the COVID-19 pandemic for the sample of adolescents studied within the PLACE-19 Study (*n* = 2448). Table S3. The results for the items included within the Factor—Convenience of the applied Food Choice Questionnaire (FCQ) for the period before and during the COVID-19 pandemic for the sample of adolescents studied within the PLACE-19 Study (*n* = 2448). Table S4. The results for the items included within the Factor—Sensory Appeal of the applied Food Choice Questionnaire (FCQ) for the period before and during the COVID-19 pandemic for the sample of adolescents studied within the PLACE-19 Study (*n* = 2448). Table S5. The results for the items included within the Factor—Natural Content of the applied Food Choice Questionnaire (FCQ) for the period before and during the COVID-19 pandemic for the sample of adolescents studied within the PLACE-19 Study (*n* = 2448). Table S6. The results for the items included within the Factor—Price of the applied Food Choice Questionnaire (FCQ) for the period before and during the COVID-19 pandemic for the sample of adolescents studied within the PLACE-19 Study (*n* = 2448). Table S7. The results for the items included within the Factor—Weight Control of the applied Food Choice Questionnaire (FCQ) for the period before and during the COVID-19 pandemic for the sample of adolescents studied within the PLACE-19 Study (*n* = 2448). Table S8. The results for the items included within the Factor—Familiarity of the applied Food Choice Questionnaire (FCQ) for the period before and during the COVID-19 pandemic for the sample of adolescents studied within the PLACE-19 Study (*n* = 2448). Table S9. The results for the items included within the Factor—Ethical Concern of the applied Food Choice Questionnaire (FCQ) for the period before and during the COVID-19 pandemic for the sample of adolescents studied within the PLACE-19 Study (*n* = 2448).
