# Supplementary material for: Population-Based Study of the Changes in the Food Choice Determinants of Secondary School Students: Polish Adolescents’ COVID-19 Experience (PLACE-19) Study
Source: Nutrients. 2020 Aug 30;12(9):2640. doi: 10.3390/nu12092640 (PMC7551462; doi:10.3390/nu12092640)
Supplement: Supplementary file 1 [file nutrients-12-02640-s001.pdf]

nutrients

Supplementary Material

Population-Based Study of the Changes in the Food Choice Determinants of Secondary School Students: Polish Adolescents' COVID-19 Experience (PLACE-19) Study

Dominika Głąbska [1,*], Dominika Skolmowska [1], and Dominika Guzek [2]

[1] Department of Dietetics, Institute of Human Nutrition Sciences, Warsaw University of Life Sciences (SGGW-WULS), 159C Nowoursynowska Street, 02-776 Warsaw, Poland; dominika_skolmowska@sggw.pl (D.S.)

[2] Department of Food Market and Consumer Research, Institute of Human Nutrition Sciences, Warsaw University of Life Sciences (SGGW-WULS), 159C Nowoursynowska Street, 02-776 Warsaw, Poland; dominika_guzek@sggw.pl (D.Gu.)

* Correspondence: dominika_glabska@sggw.pl (D.Gl.); Tel.: +48-22-593-71-34

Supplementary Table S1. The results for the items included within the Factor – Health of the applied Food Choice Questionnaire (FCQ) for the period before and during the COVID-19 pandemic for the sample of adolescents studied within the PLACE-19 Study (n = 2448).

Items included within Factor – Health	Before the COVID-19 pandemic		During the COVID-19 pandemic		*p*	*r*
	Median (IQR)	25th-75th	Median (IQR)	25th-75th		
Is high in fiber and roughage	2.0 (1.0)	2.0-3.0	2.0 (1.0)	2.0-3.0	< 0.0001	0.26
Is nutritious	3.0 (2.0)	2.0-4.0	3.0 (2.0)	2.0-4.0	0.0023	0.11
Contains a lot of vitamins and minerals	3.0 (2.0)	2.0-4.0	3.0 (2.0)	2.0-4.0	0.0034	0.10
Is high in protein	3.0 (1.0)	2.0-3.0	3.0 (2.0)	2.0-4.0	0.0051	0.11
Keeps me healthy	3.0 (2.0)	2.0-4.0	3.0 (2.0)	2.0-4.0	0.0970	0.06
Is good for my skin/teeth/hair/nails etc.	3.0 (2.0)	2.0-4.0	3.0 (2.0)	2.0-4.0	0.0064	0.11

IQR – interquartile range.

Supplementary Table S2. The results for the items included within the Factor – Mood of the applied Food Choice Questionnaire (FCQ) for the period before and during the COVID-19 pandemic for the sample of adolescents studied within the PLACE-19 Study (n = 2448).

**Items included within Factor – Mood**	**Before the COVID-19 pandemic**		**During the COVID-19 pandemic**		***p***	***r***
	**Median (IQR)**	**25th-75th**	**Median (IQR)**	**25th-75th**		
Makes me feel good	4.0 (1.0)	3.0-4.0	3.0 (2.0)	2.0-4.0	< 0.0001	0.60
Helps me cope with stress	2.0 (2.0)	1.0-3.0	2.0 (2.0)	1.0-3.0	0.0001	0.13
Keeps me awake/alert	3.0 (1.0)	2.0-3.0	3.0 (2.0)	2.0-4.0	0.2853	0.04
Helps me relax	3.0 (1.0)	2.0-3.0	2.0 (1.0)	2.0-3.0	0.1012	0.05
Cheers me up	3.0 (2.0)	2.0-4.0	3.0 (2.0)	2.0-4.0	< 0.0001	0.28
Helps me to cope with life	2.0 (2.0)	1.0-3.0	2.0 (2.0)	1.0-3.0	< 0.0001	0.15

IQR – interquartile range.

Supplementary Table S3. The results for the items included within the Factor – Convenience of the applied Food Choice Questionnaire (FCQ) for the period before and during the COVID-19 pandemic for the sample of adolescents studied within the PLACE-19 Study (n = 2448).

**Items included within Factor – Convenience**	**Before the COVID-19 pandemic**		**During the COVID-19 pandemic**		***p***	***r***
	**Median (IQR)**	**25th-75th**	**Median (IQR)**	**25th-75th**		
Is easy to prepare	2.0 (1.0)	2.0-3.0	3.0 (1.0)	2.0-3.0	< 0.0001	0.13
Is easily available in shops and supermarkets	3.0 (2.0)	2.0-4.0	3.0 (2.0)	2.0-4.0	0.9026	0.00
Takes no time to prepare	3.0 (2.0)	2.0-4.0	3.0 (2.0)	2.0-4.0	0.0002	0.12
Can be cooked very simply	3.0 (2.0)	2.0-4.0	3.0 (2.0)	2.0-4.0	0.1624	0.05
Can be bought in shops close to where I live or work	3.0 (2.0)	2.0-4.0	3.0 (2.0)	2.0-4.0	< 0.0001	0.16

IQR – interquartile range.

Supplementary Table S4. The results for the items included within the Factor – Sensory Appeal of the applied Food Choice Questionnaire (FCQ) for the period before and during the COVID-19 pandemic for the sample of adolescents studied within the PLACE-19 Study (n = 2448).

**Items included within Factor – Sensory Appeal**	**Before the COVID-19 pandemic**		**During the COVID-19 pandemic**		***p***	***r***
	**Median (IQR)**	**25th-75th**	**Median (IQR)**	**25th-75th**		
Tastes good	4.0 (1.0)	3.0-4.0	4.0 (1.0)	3.0-4.0	< 0.0001	0.51
Smells nice	3.0 (1.0)	3.0-4.0	3.0 (2.0)	2.0-4.0	< 0.0001	0.28
Has a pleasant texture	3.0 (1.0)	2.0-3.0	2.0 (1.0)	2.0-3.0	0.6345	0.02
Looks nice	3.0 (2.0)	2.0-4.0	3.0 (2.0)	2.0-4.0	< 0.0001	0.15

IQR – interquartile range.

Supplementary Table S5. The results for the items included within the Factor – Natural Content of the applied Food Choice Questionnaire (FCQ) for the period before and during the COVID-19 pandemic for the sample of adolescents studied within the PLACE-19 Study (n = 2448).

**Items included within Factor – Natural Content**	**Before the COVID-19 pandemic**		**During the COVID-19 pandemic**		***p***	***r***
	**Median (IQR)**	**25th-75th**	**Median (IQR)**	**25th-75th**		
Contains no additives	2.0 (2.0)	1.0-3.0	2.0 (2.0)	1.0-3.0	< 0.0001	0.20
Contains natural ingredients	1.0 (1.0)	1.0-2.0	3.0 (2.0)	2.0-4.0	0.1709	0.05
Contains no artificial ingredients	2.0 (2.0)	1.0-3.0	3.0 (2.0)	2.0-4.0	0.5096	0.02

IQR – interquartile range.

Supplementary Table S6. The results for the items included within the Factor – Price of the applied Food Choice Questionnaire (FCQ) for the period before and during the COVID-19 pandemic for the sample of adolescents studied within the PLACE-19 Study (n = 2448).

**Items included within Factor – Price**	**Before the COVID-19 pandemic**		**During the COVID-19 pandemic**		***p***	***r***
	**Median (IQR)**	**25th-75th**	**Median (IQR)**	**25th-75th**		
Is not expensive	3.0 (1.0)	2.0-3.0	3.0 (2.0)	2.0-4.0	0.0007	0.11
Is good value for money	3.0 (2.0)	2.0-4.0	3.0 (2.0)	2.0-4.0	< 0.0001	0.15
Is cheap	3.0 (1.0)	2.0-3.0	3.0 (2.0)	2.0-4.0	< 0.0001	0.18

IQR – interquartile range.

Supplementary Table S7. The results for the items included within the Factor – Weight Control of the applied Food Choice Questionnaire (FCQ) for the period before and during the COVID-19 pandemic for the sample of adolescents studied within the PLACE-19 Study (n = 2448).

Items included within Factor – Weight Control	Before the COVID-19 pandemic		During the COVID-19 pandemic		*p*	*r*
	Median (IQR)	25th-75th	Median (IQR)	25th-75th		
Is low in calories	2.0 (2.0)	1.0-3.0	2.0 (2.0)	1.0-3.0	< 0.0001	0.33
Is low in fat	2.0 (1.0)	2.0-3.0	3.0 (1.0)	2.0-3.0	< 0.0001	0.14
Helps me control my weight	3.0 (2.0)	2.0-4.0	3.0 (2.0)	2.0-4.0	0.0001	0.15

IQR – interquartile range.

Supplementary Table S8. The results for the items included within the Factor – Familiarity of the applied Food Choice Questionnaire (FCQ) for the period before and during the COVID-19 pandemic for the sample of adolescents studied within the PLACE-19 Study (n = 2448).

Items included within Factor – Familiarity	Before the COVID-19 pandemic		During the COVID-19 pandemic		*p*	*r*
	Median (IQR)	25th-75th	Median (IQR)	25th-75th		
Is familiar	3.0 (1.0)	2.0-3.0	3.0 (1.0)	2.0-3.0	0.6333	0.01
Is like the food I ate when I was a child	2.0 (2.0)	1.0-3.0	2.0 (2.0)	1.0-3.0	0.0086	0.09
Is what I usually eat	2.0 (1.0)	2.0-3.0	2.0 (1.0)	2.0-3.0	0.5495	0.02

IQR – interquartile range.

Supplementary Table S9. The results for the items included within the Factor – Ethical Concern of the applied Food Choice Questionnaire (FCQ) for the period before and during the COVID-19 pandemic for the sample of adolescents studied within the PLACE-19 Study (n = 2448).

**Items included within Factor – Ethical Concern**	**Before the COVID-19 pandemic**		**During the COVID-19 pandemic**		***p***	***r***
	**Median (IQR)**	**25th-75th**	**Median (IQR)**	**25th-75th**		
Is packaged in an environmentally friendly way	2.0 (2.0)	1.0-3.0	2.0 (2.0)	1.0-3.0	0.2710	0.04
Comes from countries I approve of politically	1.0 (1.0)	1.0-2.0	2.0 (2.0))	1.0-3.0	< 0.0001	0.31
Has the country of origin clearly marked	2.0 (2.0)	1.0-3.0	2.0 (2.0)	1.0-3.0	0.2595	0.04

IQR – interquartile range.

© 2020 by the authors. Licensee MDPI, Basel, Switzerland. This article is an open access article distributed under the terms and conditions of the Creative Commons Attribution (CC BY) license (http://creativecommons.org/licenses/by/4.0/).
